# Nervous and Mental Disorders

**Published:** 1918-06-15

**Authors:** Madeline A. S. Puckle


### THE EDITOR'S LETTER-BOX.

### NERVOUS AND MENTAL DISORDERS.

### Uses of Wallpaper in their Treatment.

*To the Editor of* THE HOSPITAL.

SIR,—That elusive quality known as "taste," in the selection of the colour and form with which we clothe ourselves and our environment—if allowed individual expression—conveys more or less to others an indication of our mental outlook and composition. There are certain broad lines which show trend of thought, and along which definite types of mind evolve with slight variations. Here and there, we meet with absolute originality in form and colour chosen, proclaiming an entirely different view-point from that of the generality of people, and the result is either imitated or ridiculed according to the sympathetic or negative attitude of the beholders.

Given a certain "temperament," it is not difficult to foretell on what lines the apparel and home decoration are likely to run. For instance, a person of sporting disposition, with good physique, health, and vigour, is hardly likely to dress in "flimsy" materials, "shades" instead of colours, a multiplicity of small personal adornments and details of dress, or to have her special "sanctum" arranged with a "subtle" effect and devotion to the cult of the diminutive. Rather would one expect broad effects, neatness of outline, and perhaps a tendency to slight blatancy in the matter of colour and pattern. In a person of somewhat unobtrusive disposition we should look for neutral shades and quiet effects in home surroundings. These instances may be multiplied to express all the varieties of people with whom we come in contact, and it is by such means that we can judge to some degree the mental outlook on life of the individual. Therefore, we may assume that, as personality has a decided influence on environment, environment may exercise a considerable influence on personality, and as environment includes very largely "form" and "colour," so these may play a large part in their effect on the mentality of the individual.

THE EFFECT OF COLOUR ON THE INDIVIDUAL.

It is a truth of general acceptance that certain colours have certain effects on mentality. The exciting influence of red is well known, the calming effect of pale blue, the hopeful effect of yellow and pale pink—to name only a few—and the happy or unhappy combination of some of these, cause the beholder the sensation of pleasure or the reverse, showing that emotion is affected by colour and groupings of colour, varying according to the amount of "perception" and capability of "sensation" possessed by the individual.

Equally important in its mental suggestion is *form*, and the mind cannot appreciate fully any scheme which in outline does not meet with its approval, and ugliness or want of proportion in the outline will produce a sensation of dislike, if not of irritation.

There must be a harmony of both form and colour, as well as a sense of balance and proportion, to ensure complete satisfaction to the mind's eye. If this is so in the normal individual, it must be very greatly accentuated in the case of one whose mind is *not* normal, or whose nerves are hypersensitive or disordered, and, therefore, the therapeutic value of suitable schemes of environment must be considerable in inducing a return to the normal and in avoiding conscious or subconscious irritating emotions.

FORM AS A CURATIVE AGENT.

Years ago, in an institution for the treatment of mental and nervous disorders, various coloured rooms were in use for different states and varieties of these complaints, and a similar scheme has again been put forward for the treatment of cases of "shell-shock"; but has the use of *form* as a curative agent ever been suggested in conjunction with *colour*? How much increased misery may be caused to those of deranged mental vision by the constant sight of patterns and colours, which, through "association of ideas," may suggest terrifying thoughts to their minds, or further their states of depression! They are not always able to express their feelings, but it is often said that bold designs "seem to jump at you," or that a paper is "cheerful" or "gloomy," and it is common knowledge that phases of delirium occur in which the wallpaper appears to be distorted into hideous faces, or that the patterns are "running into each other," and so on.

We know that a horizontal line suggests continuity, a perpendicular line ambition or rectitude, a line curved upwards hopefulness or rejoicing, and a downward curve despair or grief, else why do we depict the misanthrope with mouth turned down at the corners, or our festoons curved upward on occasions of rejoicing, or our churches built with spires pointing heavenwards? It is clear that the use of these forms has a general significance, and therefore they must have an influence—conscious or subconscious—on the mind, and therefore they can be of use as one of the means of bringing back the abnormal into its accustomed condition.

Cases of neurasthenia and similar disorders may be very much affected by even such an apparently unimportant matter as a jarring, ugly pattern or wrong colouring, and incipient delusions may be fostered thereby. Quite small things may have an effect on the subconscious mind, and may give rise to results at a later period. A visit to a large town by a person who lives in the country: the unaccustomed noise and bustle may or may not tire him at the time, but on returning to the quieter surroundings, a headache or eyestrain will not improbably result from the continuous sound and movement.

THE WALLPAPER FOR THE EXCITABLE.

To return to the subject of wallpapers as a curative factor. It is suggested that definite schemes of pattern and colour should be tried for different grades and types of nervous or mental disorders. For instance, for excitability: a plain, blue paper with a somewhat indefinite horizontal design and no frieze, nothing that will fix the eye or cause it to look upward; all the furnishing of the room in quiet tones without a striking pattern, and shaded lights well on the *level* of the eyes, not *above*, and blinds or curtains to the windows in harmonising tone to screen off any superfluous light.

For depression: a wallpaper of light tone with small pink or yellow-hued festoons or sprays of rose-pink flowers indefinitely grouped, with a frieze of brighter shade; a dark carpet, and the whole tone of the room to be lighter and brighter towards the ceiling than on the level of the eyes, and any pictures hung "above the line," to induce a tendency to look *upward* and *forward*.

For delusional cases where self-accusations are prominent: a plain paper of light tone, such as a deep cream, with satin stripes running up to a frieze of a pastoral or simple flowered design, giving an impression of rectitude and return to innocence; the carpet of a moss green, and touches of subdued but cheerful patterns in the furnishing, with plenty of light from the windows to give the whole a hopeful impression.

All bedrooms should be as *restful* in design as possible without being insipid, and no bedstead should face the window. In larger institutions, where there are many patients of various degrees of illness, it is not possible perhaps to do more than colour-wash or paint the walls one shade, and in this case the safest and most durable tones are pale lemon yellow and pale pinkish terra-cotta. They do not fade or discolour as do greens and blues, and, taking it all round, are suitable for the majority of cases.—Yours faithfully, MADELINE A. S. PUCKLE.

